# Performance Evaluation of Carrier-Frequency Offset as a Radiometric Fingerprint in Time-Varying Channels

**DOI:** 10.3390/s24175670

**Published:** 2024-08-31

**Authors:** Abdulsahib Albehadili, Ahmad Y. Javaid

**Affiliations:** 1 Department of Electrical Engineering and Computer Science, The University of Toledo, Toledo, OH 43606, USA; abdulsahib.albehadili@utoledo.edu; 2 Department of Computer Engineering Technology, College of Information Technology, Imam Ja’afar Al-Sadiq University, Najaf 54001, Iraq

**Keywords:** carrier frequency offset (CFO), machine learning (ML), orthogonal frequency-division multiplexing (OFDM), PHY-authentication, software-defined radio (SDR)

## Abstract

The authentication of wireless devices through physical layer attributes has attracted a fair amount of attention recently. Recent work in this area has examined various features extracted from the wireless signal to either identify a uniqueness in the channel between the transmitter–receiver pair or more robustly identify certain transmitter behaviors unique to certain devices originating from imperfect hardware manufacturing processes. In particular, the carrier frequency offset (CFO), induced due to the local oscillator mismatch between the transmitter and receiver pair, has exhibited good detection capabilities in stationary and low-mobility transmission scenarios. It is still unclear, however, how the CFO detection capability would hold up in more dynamic time-varying channels where there is a higher mobility. This paper experimentally demonstrates the identification accuracy of CFO for wireless devices in time-varying channels. To this end, a software-defined radio (SDR) testbed is deployed to collect CFO values in real environments, where real transmission and reception are conducted in a vehicular setup. The collected CFO values are used to train machine-learning (ML) classifiers to be used for device identification. While CFO exhibits good detection performance (97% accuracy) for low-mobility scenarios, it is found that higher mobility (35 miles/h) degrades (72% accuracy) the effectiveness of CFO in distinguishing between legitimate and non-legitimate transmitters. This is due to the impact of the time-varying channel on the quality of the exchanged pilot signals used for CFO detection at the receivers.

## 1. Introduction

The seamless connection offered by wireless technology is envisioned to cover wider domains, where the physical and digital worlds can merge. Such integration will provide new possibilities and applications where human-to-machine and machine-to-machine interactions will deliver more efficient and sustainable experiences. This, however, necessitates advancements in security methods to ensure reliability, especially with the open and broadcast nature of wireless channel, where transmissions from communicating parties are more exposed to eavesdropping. While legacy security methods offered by the top layers of the protocol stack attempt to provide a data CIA triad (confidentiality, integrity, and availability), nonetheless, they were initially devised with assumptions of high computational power availability for the communicating parties. This, however, is not always the case, especially with the proliferation of low-power wireless devices with limited resources. Physical layer security (PLS), on the other hand, exploits physical layer attributes to provide an additional level of data security. PLS is envisioned to either act as a standalone security layer for low-power devices that cannot afford to implement legacy security approaches, or it can be incorporated as an additional security layer alongside legacy approaches [1]. Various research lines have looked closely at PLS to provide the CIA triad [2,3,4]. The first line has devised PHY-encryption methods to maintain reliable communication at authorized receivers, while equivocating data recovery at unauthorized receivers by, for instance, making received signals noisy or contaminated only for unauthorized parties [2]. The second line has exploited shared randomness of the wireless channel (e.g., the reciprocal channel impulse response (CIR)) between legitimate parties to extract symmetric secret keys. The privacy of such extracted keys is based on the premise that the CIR is spatially decorrelated for unauthorized parties located half of the signal wavelength apart from authorized ones, ending up with dissimilar extracted keys [3]. The third line, which is the focus of this work, has exploited PHY-layer features to provide PHY-authentication. This is achieved through extracting unique identifiers, termed radio frequency fingerprints (RFFs), to distinguish between different transmitters [4]. RFF features can be extracted from received wireless signals to either define the uniqueness in the common channel between the transmitter-and-receiver pair [5], or to find certain unique hardware behaviors that can be exploited to identify a specific transmitter [6]. The latter has been found to be more robust, as it is hardware-based and exhibits better stability as opposed to channel-based methods that can be impacted by dynamic channel behaviors. With imperfect hardware manufacturing processes, it is almost impossible to fine-tune transceiver components (such as local oscillators (LO)) to exhibit similar performances for different transceivers. This creates intrinsic behaviors that can uniquely identify wireless devices. LO mismatch, for instance, in a specific transmitter-and-receiver pair induces a carrier frequency offset (CFO) that is different from other transceiver pairs. From a PLS perspective, this CFO is desirable and can be exploited to differentiate between different transmitters [6]. Since CFO estimation is an indigenous process that has always been an essential part of the receiver block chain, no extra processing is required for its extraction, which makes it an attractive hardware-specific RFF feature for PHY-authentication. CFO has been utilized, for instance, to detect rogue WiFi transmission for smartphones [7]. In [8], frequency and phase differences were extracted from the QPSK constellation to identify Four Zigbee devices for a USRP2 receiver. The work in [9] conducted a study of 93 Wi-Fi devices with 13 different models. Besides the CFO, sampling frequency offset, transmitter turn on/off transients, and scrambling seed features were utilized for device classification. In addition, the work in [10] used the CFO to correct IQ signals, before using them to train CNN classifiers, where a spectrogram-based analysis was conducted to extract the received signal time–frequency characteristics of 20 LoRA devices with a detection accuracy of 97.6%. While the works in [7,8,9,10,11] showed great effectiveness in identifying wireless transmitters with CFO; nonetheless, only stationary transmission scenarios were examined. The work in [6] investigated CFO performance in time-varying mobile channels, where a Kalman filter was adopted to refine estimated time-variant CFO values before directly using them for device identification. Nonetheless, the CFO values were simulated as an autoregressive random process rather than being extracted from real-world transmission in a mobility environment. In our previous work [12], CFO values were collected in a moderate mobility scenario, which showed a detection performance of 99% (with 0.4% false alarm rate) for walking speed and 98% (0.6%) for a 10 miles/h driving speed. Nonetheless, with higher speed scenarios, it is still unclear whether CFO would exhibit the stability to be used for PHY-authentication. Furthermore, while LO mismatch is a major and desired contributor to CFO, there are other detrimental factors that can impact estimated CFO values at the receiver, such as the Doppler effect (induced by motion) and time-varying channels [13]. The Doppler effect can be predictable, as its only source is motion. The time-varying channel factor, however, is stochastic and unpredictable, which can affect the pilot symbols (incorporated in the preambles of transmitted signals) used for CFO estimation. This induces instability in the estimated CFO values, degrading their quality as an RFF source for PHY-authentication. Doppler shift and time-varying channel effects are coupled together when detecting CFO at the receiver. While outside the scope of our current work, there are methods that can adopted to resolve such coupling. For instance, the work in [14] devised a unified framework for joint channel and target (physical) parameter estimation, which is based on canonical polyadic decomposition (CPD). Such CPD characterization allows for the estimation of the angle of arrival/departure (AoA/AoD), time delay, and Doppler shift in a separate manner. It uses an iterative estimation (optimizing the underlying parameters in a sequential manner) method to address the coupling between AoD and Doppler shift parameters. Such insight can be used to resolve the coupling between the channel taps and the Doppler effect when estimating the CFO. In [15], channel estimation was improved by dividing the estimation process into two separate stages: the AoA/AoD estimation stage, and the channel tap estimation stage. The rationale was that AOA/AoD vary much more slowly than the channel taps. This observation can be exploited to make the estimation process more efficient and accurate, as the AOA/AoD estimated in the first stage can be used to improve the estimation of the channel taps in the second stage. Such a rationale can also be used to improve the accuracy of CFO estimation, where the Doppler effect can be estimated first and then utilized to improve the estimation of the channel taps.

In this work, we investigate the performance of CFO for PHY-authentication in time-varying channels. The contributions of this work can summarized as following:

- A software-defined radio (SDR) platform is implemented to extract CFO values in a vehicular setup with mobility. A custom implementation is added to the OFDM transceiver to extract CFO values from pilot signals exchanged between the transmitter and receiver. This allows for the investigation of CFO values in realistic scenarios, instead of relying on simulation generated values as in previous studies.
- Higher mobility scenarios are explored to investigate the validity of CFO as a radio-frequency fingerprint for PHY-authentication when the channel is more dynamic.
- Machine learning (ML) classifiers are adopted to be trained and tested on the extracted CFO values for PHY-authentication. Different from conventional approaches that rely on model-based statistical signal processing for classification, which are built with assumptions and designed for inference about the relationships between random variables to estimate one variable from another observation variable, ML approaches are data driven and can adapt to various scenarios with mild assumptions about the environments studied.

The rest of this paper is organized as follows: section two presents the system model, section three presents the experimental setup and results, and finally the conclusions are presented in section four.

## 2. System Model

**Figure 1:**
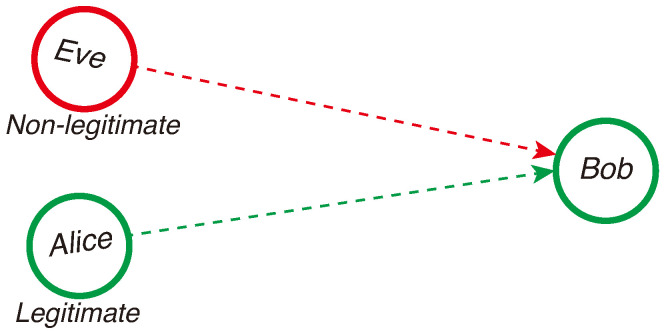
The system model where Alice and Bob are, respectively, the legitimate transmitter and receiver, while Eve is a spoofing transmitter impersonating Alice.

The system model is illustrated in Figure 1, where a legitimate transmitter, Alice, is sending messages to a legitimate receiver, Bob, over the wireless broadcast channel. At the same time, there is a non-legitimate transmitter, Eve, who is capable of impersonating Alice by replaying her transmission. Conventionally, Bob would resort to upper layer protocols to validate Alice’s transmission. Nonetheless, with PLS methods, the premise is rather to rely on RFF for authentication. Here, Bob relies on the CFO as a hardware fingerprint to distinguish between Alice’s and Eve’s transmissions. If Bob can establish an extracted CFO from a signal S(t) as Alice’s identity, he can later extract CFO from future transmissions S(t+T) to verify whether the transmission has originated from Alice. This is based on the premise that the CFO extracted from one transmitter should exhibit small variances and, as such, any abrupt changes in the estimated CFO can be attributed to a spoofing behavior, as it could be originated from different transmitter hardware, i.e., Eve. In other words,

(1a) $$\begin{array}{cc} Alice\to Bob:S(t+T) & \equiv S(t) \end{array}$$

(1b) $$\begin{array}{cc} Eve\to Bob:S(t+T) & ≢S(t) \end{array}$$

**Figure 2:**
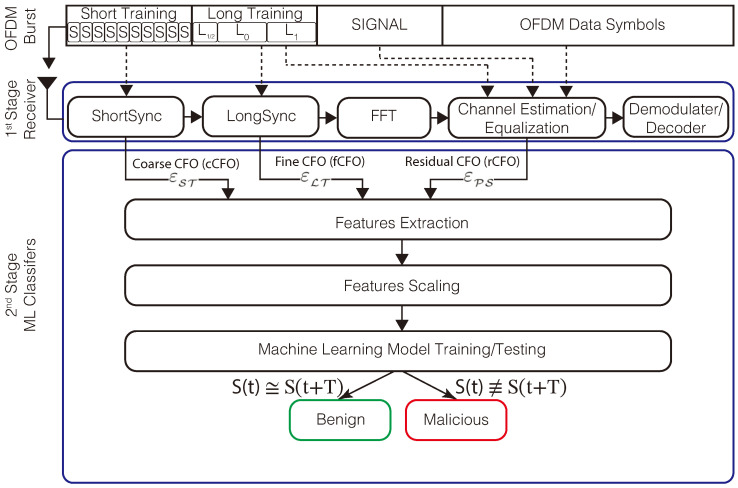
The CFO estimation and detection scheme, the top part depicts the OFDM burst which includes the ST field, LT field, and the pilot subcarriers used for CFO estimation. The first stage involves the cCFO, fCFO, and rCFO estimation from the OFDM burst. The second stage illustrates how the three extracted features are utilized for the ML classifiers training/testing.

While the assumption in Equations (1a) and (1b) is true for CFO values induced by hardware LO mismatches, there are other factors such as the Doppler effect and time-varying channel that could negatively contribute to the CFO extracted in mobility scenarios. As such, to account for these factors, we conducted experiments to extract CFO values at high speeds from incoming OFDM bursts by adopting an IEEE 802.11a/g/p transceiver [16]. Each OFDM burst, as depicted in Figure 2, contains training sequences known to the transmitter and receiver. These sequences are incorporated in three different fields; namely, short training (ST) preamble, long training (LT) preamble, and pilot subcarriers, which are, respectively, used to estimate the coarse CFO (cCFO), fine CFO (fCFO), and residual CFO (rCFO) [17]:

(2a) $$\begin{array}{cc} \varepsilon_{\mathrm{ST}} & =\frac{\vartheta }{2\times 16\pi f_{c}}∠\left(\sum_{n=0}^{N_{\mathrm{ST}}-1-16}S_{\mathrm{ST},n}^{*}\;S_{\mathrm{ST},n+16}\right) \end{array}$$

(2b) $$\begin{array}{cc} \varepsilon_{\mathrm{LT}} & =\frac{\vartheta }{2\times 64\pi f_{c}}∠\left(\sum_{n=0}^{64-1}S_{\mathrm{LT},n}^{*}\;S_{\mathrm{LT},n+64}\right) \end{array}$$

(2c) $$\begin{array}{cc} \varepsilon_{\mathrm{PS}} & =\frac{\vartheta }{2\pi f_{c}}∠\left(\sum_{n=-21,-7,7,21}S_{l,n}\;Q_{n}^{*}\right) \end{array}$$

For the cCFO estimation in Equation (2a), a 10 times repeating sequence of 16 complex (I/Q) samples in the ST field are utilized. Where $S_{\mathrm{ST},n}$ is the *n*th complex sample; $N_{\mathrm{ST}}$ is the ST sequence length (i.e., 160 samples); ϑ is the sampling rate; and $f_{c}$ is the carrier frequency. $S_{\mathrm{ST},n}$ should equal $S_{\mathrm{ST},n+16}$ due to ST sequence periodicity, and the product of $S_{\mathrm{ST},n}$ complex conjugate and $S_{\mathrm{ST},n+16}$ should yield a real number. This means when there is the presence of a CFO, a phase difference ∠(.) accumulated over $N_{\mathrm{ST}}$ samples will arise. The estimated cCFO (i.e., $\varepsilon_{\mathrm{ST}}$) is used to correct the LT sequence, i.e., $S_{\mathrm{LT},n}=S_{\mathrm{LT},n}\;\;e^{-j2\pi n\varepsilon_{\mathrm{ST}}f_{c}/\vartheta };n=32,34,\ldots ,159$. Following LT sequence correction, the $L_{0}$ and $L_{1}$ in the LT field are utilized for fCFO ($\varepsilon_{\mathrm{LT}}$) estimation in Equation (2b). Where $S_{\mathrm{LT},n}$ is the $n^{th}$ complex sample. $L_{1/2}$, $L_{0}$, and $L_{1}$ make up 160 samples in total. $L_{1/2}$ encompasses 32 samples and is used as a guard interval. $L_{0}$ and $L_{1}$, on the other hand, are identical and each contains 64 complex samples. Finally, the frequency offset not compensated through cCFO and fCFO corrections is captured through the rCFO, which is estimated in Equation (2c) with four pilot subcarriers in each OFDM symbol following the preamble fields. Starting at the SIGNAL field, l=1,2,… is the OFDM symbol index; $Q_{n},n\in \left\{\pm 21,\pm 7\right\},$ are the corresponding channel gains estimated earlier through the LT sequence. Here, each OFDM symbol contains 64 subcarriers with only 52 utilized, while for the remaining 12 null subcarriers, one is used as a DC subcarrier to suppress LO leakages and 11 are used as guard intervals to minimize inter-symbol interference (ISI). Out of the 52 subcarriers, only 48 are used to carry the data payload, and the remaining four (index: ±7, ±21) encompass pilots for rCFO estimation.

**Figure 3:**
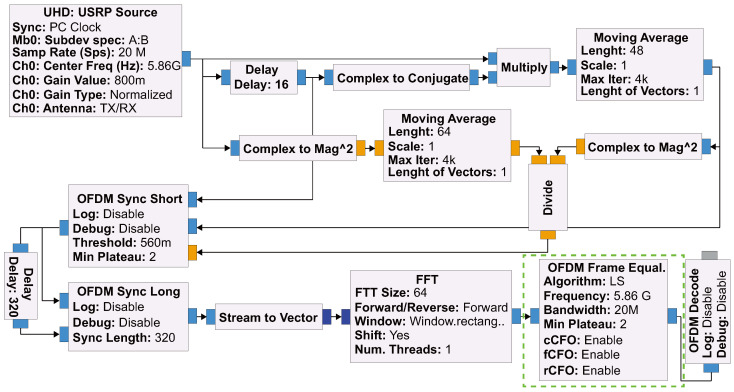
The GNURadio flowgraph of the OFDM receiver. The OFDM frame equalizer block (in dashed green line) includes our added code to extract cCFO, fCFO, and rCFO values estimated from received OFDM bursts detected in the flowgraph blocks preceding the equalizer block.

The estimated CFO values in Equations (2a)–(2c) are used as RFF features, which comprise the first stage of the detection scheme in Figure 2. This was realized with an SDR testbed that consisted of a GNURadio software implementation of OFDM-based transceiver (complying with IEEE 802.11a/g/p recommendations [16]), Figure 3, and USRP B210 hardware from Ettus research, as well as HackRF hardware. GNURadio enables one to develop and deploy real-world radio systems. It is a modular (flowgraph oriented) framework that supports DSP development in C++ and Python. It includes libraries of DSP blocks that can be readily incorporated (such as FFT block) in more complex DSP applications. In addition, it allows modification and/or development of custom blocks. The UHD block is the interface between the USRP hardware and the flowgraph, which downstreams the received RF signals as a complex baseband IQ sample-stream to the flowgraph. The flexibility offered by GNURadio allowed us to incorporate our custom logic on top of the OFDM equalizer block to extract the cCFO, fCFO, and rCFO values estimated from the received OFDM bursts detected in the flowgraph blocks preceding the equalizer block.

The extracted features were later used to train/test ML classifiers in the second stage of the detection scheme in Figure 2. Each ML classifier was implemented as a function that maps the three features Equations (2a)–(2c) into two unique classes, namely: Alice and Eve.

(3) $$\begin{array}{cc} H_{n} & =F(\varepsilon_{\mathrm{ST}}^{\left(k\right)},\varepsilon_{\mathrm{LT}}^{\left(k\right)},\varepsilon_{\mathrm{PS}}^{\left(k\right)}) \end{array}$$

where $H_{n}$ is the transmitter identifier, n∈{Alice,Eve}, F(.) is a function that maps the features Equations (2a)–(2c) into $H_{n}$, which can be any of many classification functions trained on K samples (not to be confused with the IQ samples of OFDM symbols). Out of various ML classifiers [18], we adopted four different classifiers: logistic regression (LR), k-nearest neighbors (KNN), decision tree (DT), and support vector machine (SVM). They were realized with a Python-based library that provided support for various supervised/unsupervised ML algorithms [19]. While a conventional approach for communications systems research is to rely on model-based statistical signal processing for classification. Such statistical models are built with assumptions and designed for inference about the relationships between random variables, i.e., to estimate one variable from another variable observation. Nonetheless, recent advancements in computation have opened the door for researchers to adopt ML classifiers, which are data-driven and can adapt to various scenarios with mild assumptions about the environments studied [20].

## 3. Experiments and Results

**Figure 4:**
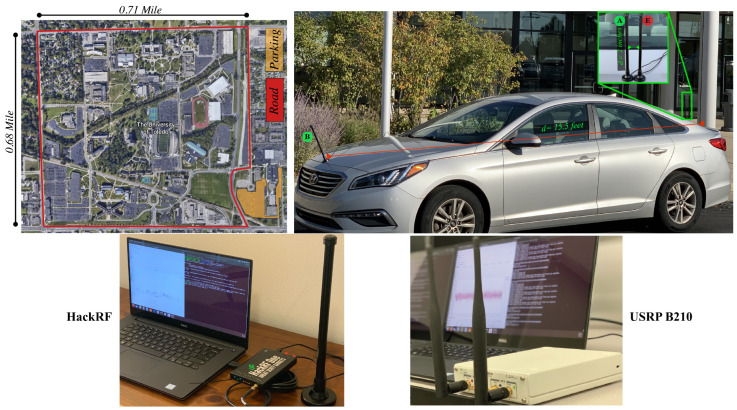
The experiment setup: Alice’s and Eve’s antennas are mounted on the rear end of the vehicle, 2 inches apart; while Bob’s antenna is mounted on the front end of the vehicle with a distance of 15.5 feet from Alice and Eve.

To evaluate the effect of a time-varying channel on CFO stability, real CFO measurements were collected in an outdoor environment on the road near the college of the engineering campus at the university of Toledo, as depicted in Figure 4. We used Dell Precision 5520 laptops (Intel Core i7-7820HQ CPU, 32 GB RAM), running Ubuntu 18.04 OS. Two USRPs (B210 model) from the same vendor were configured to be the transmitters Alice and Eve, and one HackRF was configured as the receiver. Using transmitters from the same vendor allowed us to investigate the worst-case scenario, which is when there is no significant difference between the estimated CFO values from Alice and Eve, undermining CFO’s uniqueness for identification. The experiment operation parameters are summarized in Table 1. To demonstrate a replay attack, both USRPs were configured to send the exact same messages at the same transmission rate (2 bursts/s). While the CFO is meant to capture the frequency drift due to the hardware mismatch between the transmitter and receiver, the Doppler effect caused by motion is added to the estimated CFO values at the receiver, which should also be captured and corrected with the help of the CFO estimation process. To focus on the CFO caused by the hardware mismatch, all devices and antennas were mounted on the same vehicle (rather than having two vehicles one trailing the other) to ensure that signals from both transmitters Alice and Eve would encounter the same Doppler effect, as they would be traveling at the exact same speed. This ensured the frequency offset originating from the Doppler effect would be the same for the Alice–Bob and Eve–Bob channels, eliminating its effect on the classification bias of the ML classifiers. This helped us to steer our analysis towards the effect of the time-varying channel that disturbed the pilot tones used for CFO estimation.

**Figure 5:**
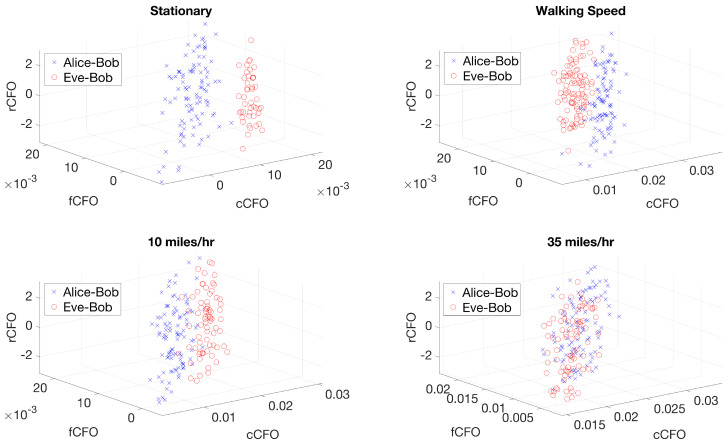
A snippet of the first 100 samples of the collected cCFO, fCFO, and rCFO values for stationary and mobility scenarios.

Alice’s and Eve’s antennas were mounted on the rear end of the vehicle, 2 inches apart; while Bob’s antenna was mounted on the front end of the vehicle, with a distance of 15.5 feet from Alice and Eve. The vehicle was driven at an average speed of 35 miles/h for the most part, and 2221 OFDM bursts were detected from each transmitter (4442 in total for two transmitters) and their corresponding CFO estimates (i.e, cCFO, fCFO, and rCFO) were extracted. The first 100 samples of the collected cCFO, fCFO, and rCFO are depicted in Figure 5 for stationary and mobility scenarios. It can be clearly observed that when there was no mobility present, the cCFO, fCFO, and rCFO values from Alice–Bob and Eve–Bob could be linearly separated. This is due to the fact that the only source of frequency offset was the hardware discrepancies between Alice’s and Eve’s transmitters. However, when there was mobility present, the detected cCFO, fCFO, and rCFO values started to overlap, especially at higher speeds (i.e., 35 miles/h), which was caused by the time-varying channel effect on the estimated cCFO, fCFO, and rCFO values at Bob.

The collected cCFO, fCFO, and rCFO were used to train/test four ML classifiers: LR, KNN, DT, and SVM. For each classifier, a κ-fold cross-validation was performed by randomly splitting the entire dataset (i.e., 4442 samples) into κ subsets (κ−1 subsets were employed for training and one for testing). This offered an unbiased classifier evaluation by ensuring each data sample was utilized for training and testing through κ iterations, demonstrating whether discrepancies in the data samples impacted the classifier performance. It is important to mention there is no rule of thumb to decide the exact number for the samples size needed for training an ML classifier; nonetheless, one factor that can be considered is the number of features (dependable variables) used. Since, in our case, the used features were only three, 2221 OFDM bursts from each transmitter should suffice.

The prediction capability of each classifier was evaluated in terms of the receiver operating characteristic (ROC) curve with its corresponding area under the curve (AUC), as well as a confusion matrix. An ROC curve depicts the probabilities of the true positives $P_{TP}$ against false positives $P_{FP}$:

(4a) $$\begin{array}{cc} P_{TP} & =P({\hat{H}}_{Alice}|H_{Alice}) \end{array}$$

(4b) $$\begin{array}{cc} P_{FP} & =P({\hat{H}}_{Eve}|H_{Alice}) \end{array}$$

**Figure 6:**
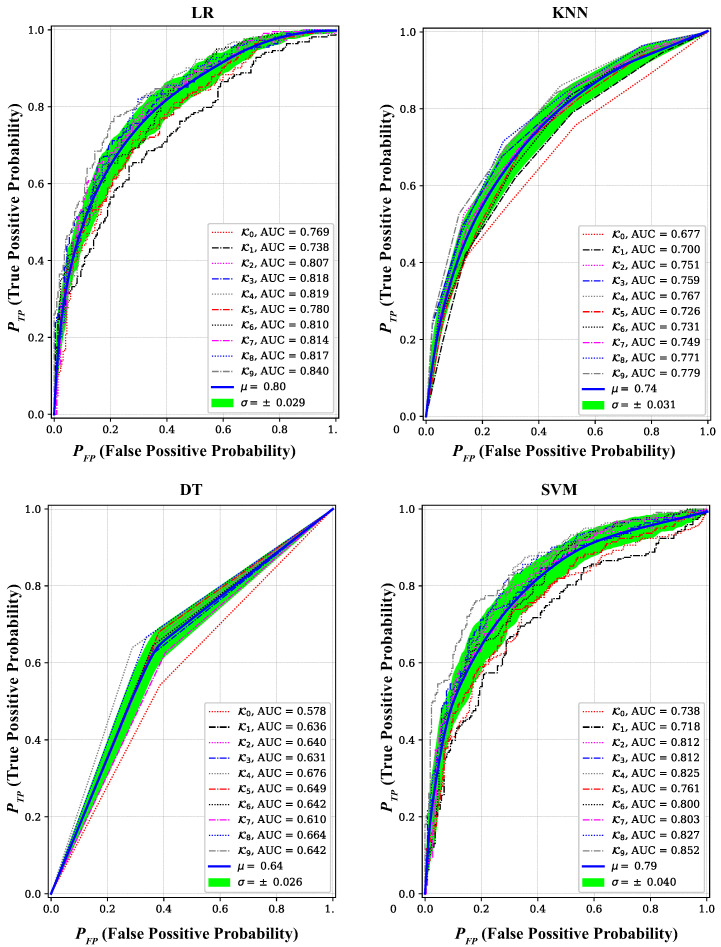
ROC curves with their corresponding area under the curve (AUC) for four ML classifiers: LR, KNN, DT, and SVM trained and tested on cCFO, fCFO, and rCFO values extracted at 35 mph speed. For κ=10 cross-validation, 10 ROC curves for each classifier with their corresponding AUCs are obtained. A higher classier performance is indicated when an ROC curve approaches the left-top corner (i.e., $P_{TP}\approx 1$ and $P_{FP}\approx 0$), accumulating larger AUC.

By setting κ=10, we obtained 10 ROC curves for each classifier with their corresponding AUCs, as depicted in Figure 6. A better classier performance is indicated when the ROC curve approaches the left-top corner (i.e., $P_{TP}\approx 1$ and $P_{FP}\approx 0$), accumulating larger AUC. It can be observed that the average AUC μ (and the standard deviation σ) for LR, KNN, DT, and SVM, respectively, were 0.8 (0.029), 0.74 (0.031), 0.64 (0.026), and 0.79 (0.04). This shows a performance degradation for the higher mobility scenario compared to cases when the car was driven at an average speed of 10 miles/h and the walking speed in our previous study [12], as summarized in Table 2.

After each classifier has been adaptively optimized with a threshold to yield the desired trade-off between true positive rate (TPR)/false positive rate (FPR), a confusion matrix could be generated to collect the classification records for all classifiers, as summarized in Table 3. For κ=10, each classifier was evaluated with 10 confusion matrices, averaged (standard deviation also found) to give the final matrix. Each matrix contains the records of the true positives (TP:Alice|Alice), true negatives (TN:Eve|Eve), false positives (FP:Eve|Alice), and false negatives (FN:Alice|Eve). This allowed calculating the TPR and FPR as follows: TPR=(TP)/(TP+FN) and FPR=(FP)/(FP+TN). Accordingly, we found the pairs of (TPR, FPR) for LR, KNN, DT, and SVM, respectively, which were (0.72, 0.27), (0.67, 0.31), (0.64, 0.36), and (0.71, 0.25). This shows a degradation in the CFO detection performance when used in higher mobility scenarios compared to lower mobility scenarios, as summarized in Table 3. This is due to the fact that in higher speed mobility scenarios, the channel becomes more dynamic and affects the values of the training pilots, which reduces the accuracy of the CFO estimation at the receiver. This shows that CFO is highly impacted by a time-varying channel, which compromises its validity for PHY-authentication. It can also be observed that the LR classifier exhibited better performance compared to the other three classifiers used. This can be attributed to the limited size of the dataset used, with a small number of features (only three features). The underlying linearity assumption between the features and the outcome variable also contributed to LR’s robustness to noise and outliers, making it less likely to overfit compared to more complex models, as it can generalize better. While the SVM was tuned with a linear kernel function, it is sensitive to hyper-parameter selection when dealing with smaller datasets with a limited number of features.

## 4. Conclusions

The performance of carrier frequency offset (CFO) for PHY-authentication was examined in a time-varying channel. While CFO is commonly considered as a solid RFF feature due to its dependence on hardware, its stability can be impacted by mobility. The pilot symbols used for CFO estimation are affected by the time-varying channel, which in turn impacts the accuracy of the estimated CFO. This decreases its stability and reliability as an RFF feature for wireless device identification. This was experimentally observed by deploying a software-defined radio (SDR) testbed to collect CFO values in a real environment, where real transmission and reception were conducted in a vehicular setup with mobility. CFO values were extracted at three levels from incoming OFDM bursts to capture the unique hardware behavior of different transmitters, namely coarse CFO, fine CFO, and residual CFO. The captured CFO values were used to train and test four classification models, namely logistic regression (LR), k-nearest neighbors (KNN), decision tree (DT), and support vector machine (SVM). The results showed that with higher mobility (35 miles/h speed), the identification performance degraded drastically. It was found that that the true positive rate (TPR) and false positive rate (FPR) for the four classifiers adopted were LR (TPR=0.72, FPR=0.27), KNN (TPR=0.67, FPR=0.31), DT (TPR=0.64, FPR=0.36), and SVM (TPR=0.71, FPR=0.25). This shows a drastic degradation in performance when compared to the 10 mile/h speed LR (TPR=0.92, FPR=0.07), KNN (TPR=0.92, FPR=0.07), DT (TPR=0.89, FPR=0.08), SVM (TPR=0.93, FPR=0.06); and walking speed LR (TPR=0.97, FPR=0.02), KNN (TPR=0.96, FPR=0.03), DT (TPR=0.95, FPR=0.04), SVM (TPR=0.97, FPR=0.02).

## Figures and Tables

**Table 1:** Experiment operational parameters.

Parameter	Quantity
Frequency	5.86 GHZ
Bandwidth	20 MHz
Transmission Mode	Half Duplex
${\mathrm{USRP}}_{1}$ RF board A: TX/RX port	Alice’s Antenna
${\mathrm{USRP}}_{2}$ RF board A: TX/RX port	Eve’s Antenna
HackRF RF board: TX/RX port	Bob’s Antenna
Antenna Type	ECOM9-5500 (9 dBi dipole)
TX Power	20 dBm
RX Gain	29 dB (i.e., 92% of maximum gain)
Channel Estimator	Least Squares
Burst Rate	2 burst/s

**Table 2:** Average area under the curve (AUC) for four ML classifiers: LR, KNN, DT, and SVM.

Speed	Avg. AUC (Std. Dev.)	LR	KNN	DT	SVM
35 mph	μ (σ)	0.8 (0.029)	0.74 (0.031)	0.64 (0.026)	0.79 (0.04)
10 mph [12]		0.97 (0.007)	0.97(0.012)	0.91 (0.031)	0.98(0.006)
Walking [12]		0.99 (0.004)	0.99 (0.009)	0.96 (0.018)	0.99(0.004)

**Table 3:** Confusion matrices for four ML classifiers: LR, KNN, DT, and SVM found for mobility scenarios of 35 miles/h, 10 miles/h, and walking speed.

35 mph	LR (TPR = 0.72, FPR = 0.27)			KNN (TPR = 0.67, FPR = 0.31)			DT (TPR = 0.64, FPR = 0.36)			SVM (TPR = 0.71, FPR = 0.25)		
		Alice	Eve		Alice	Eve		Alice	Eve		Alice	Eve
	Alice	162 (6)	60 (5)	Alice	155 (5)	67 (5)	Alice	141 (7)	81 (7)	Alice	168 (6)	54 (6)
	Eve	62 (10)	160 (10)	Eve	74 (11)	148 (11)	Eve	79 (7)	143 (6)	Eve	67 (12)	155 (12)
10 mph	LR (TPR = 0.92, FPR = 0.07)			KNN (TPR = 0.92, FPR = 0.07)			DT (TPR = 0.89, FPR = 0.08)			SVM (TPR = 0.93, FPR = 0.06)		
		Alice	Eve		Alice	Eve		Alice	Eve		Alice	Eve
	Alice	250 (4)	20 (4)	Alice	251 (4)	19 (4)	Alice	274 (5)	23 (5)	Alice	254 (5)	16 (5)
	Eve	23 (15)	247 (15)	Eve	22 (15)	248 (15)	Eve	28 (20)	242 (20)	Eve	19 (15)	251 (15)
Walking	LR (TPR = 0.97, FPR = 0.02)			KNN (TPR = 0.96, FPR = 0.03)			DT (TPR = 0.95, FPR = 0.04)			SVM (TPR = 0.97, FPR = 0.02)		
		Alice	Eve		Alice	Eve		Alice	Eve		Alice	Eve
	Alice	195 (4)	5 (4)	Alice	194 (4)	6 (4)	Alice	192 (6)	8 (6)	Alice	195 (4)	5 (4)
	Eve	6 (5)	194 (5)	Eve	7 (5)	193 (5)	Eve	9 (6)	191 (6)	Eve	6 (5)	194 (5)

## Data Availability

The datasets generated and analyzed during the current study are available from the corresponding author on reasonable request.
